# Computational perivascular space volumes derived from magnetic resonance imaging relate to lower cognitive performance and cognitive decline ‐ A multicentre individual patient data meta‐analysis

**DOI:** 10.1002/alz70856_099155

**Published:** 2025-12-25

**Authors:** Francesca M Chappell, Roberto Duarte, Maria del C. Valdes Hernandez, Rosalind Brown, Jose Bernal, Lucia Ballerini, Joel Ramirez, Stephanie Berberian, Lauren Abby Woods, Hugo Kuijf, Alberto De Luca, Geert Jan Biessels, Sandra E. Black, Joanna M Wardlaw

**Affiliations:** ^1^ Centre for Clinical Brain Sciences, University of Edinburgh, Edinburgh, Scotland, United Kingdom; ^2^ Centre for Clinical Brain Sciences, The University of Edinburgh, Edinburgh, Scotland, United Kingdom; ^3^ Centre for Clinical Brain Sciences, University of Edinburgh, Edinburgh, United Kingdom; ^4^ Institute of Cognitive Neurology and Dementia Research (IKND), Otto‐von‐Guericke University, Magdeburg, Germany; ^5^ German Center for Neurodegenerative Diseases (DZNE), Magdeburg, Germany; ^6^ University for Foreigners of Perugia, Perugia, Italy; ^7^ Dr. Sandra E. Black Centre for Brain Resilience and Recovery, LC Campbell Cognitive Neurology, Hurvitz Brain Sciences Program, Sunnybrook Research Institute, University of Toronto, Toronto, ON, Canada; ^8^ University of Toronto Scarborough, Toronto, ON, Canada; ^9^ UMC Utrecht Brain Center, Utrecht, Netherlands; ^10^ University Medical Center Utrecht, Utrecht, Netherlands; ^11^ UMC Utrecht Brain Center, University Medical Center Utrecht, Utrecht, Netherlands; ^12^ University of Toronto, Toronto, ON, Canada; ^13^ Sunnybrook Health Sciences Centre, University of Toronto, Toronto, ON, Canada; ^14^ Sunnybrook Health Sciences Centre, Toronto, ON, Canada; ^15^ UK Dementia Research Institute, University of Edinburgh, Edinburgh, Scotland, United Kingdom; ^16^ Edinburgh Imaging, Edinburgh, United Kingdom

## Abstract

**Background:**

Perivascular spaces (PVS), when enlarged, become visible and quantifiable on brain magnetic resonance images (MRI). PVS visibility in MRI has been found to be associated with ageing, hypertension, sleep disorders, and cerebral small vessel disease, but their relationship with cognition in adults remains unclear.

**Method:**

Here, we aimed to determine whether PVS volumes were associated with cognitive performance, using MRI and cognitive data from 14 different cohorts on ageing, dementia, and cerebrovascular disease. We computationally segmented PVS and estimated PVS volumes within the basal ganglia (BG‐PVS) and cerebral white matter, primarily including the centrum semiovale (CSO‐PVS) regions‐of‐interest. We then performed cross‐sectional and longitudinal meta‐analyses, both accounting for random effects. The cross‐sectional meta‐analysis employed a linear mixed model to relate PVS volumes to cognitive performance scores (Montreal Cognitive Assessment, MoCA or, Toronto Cognitive Assessment, TorCA) divided by the maximum in each cohort. The longitudinal meta‐analysis used logistic regression to predict cognitive status, either remaining cognitively normal or developing impairment over time, from PVS volumes as a percentage in the respective regions‐of‐interest.

**Result:**

*Cross‐sectional meta‐analysis* (*n* = 17771). Individuals with higher fractional PVS volumes had lower cognitive performance scores. This observation was consistent across multiple studies and remained significant after adjusting for age, sex, white matter hyperintensities, and years of education. A 10% change in CSO‐PVS volume would estimate a 2% reduction in cognitive score. *Longitudinal meta‐analysis* (*n* = 2447). Higher fractional PVS volumes tended to predict a greater likelihood of any cognitive impairment, although this relationship varied according to the participant populations (healthy aging, stroke, memory clinic) and/or other long‐term brain changes (e.g., atrophy, worsening WMH), and variation in duration of follow‐up.

**Conclusion:**

The volume of MRI‐visible PVS impacts ageing, vascular or neurogenerative processes leading to cognitive decline. More longitudinal data are needed to confirm the association. Other PVS measures should be assessed as these may be more sensitive to cognitive decline.

